# *Notes from the Field:* Interpretation of Rapid Diagnostic Tests for Leptospirosis During a Dengue Outbreak — Yap State, Federated States of Micronesia, 2019

**DOI:** 10.15585/mmwr.mm6948a6

**Published:** 2020-12-04

**Authors:** Patrick Dawson, Maria Marfel, Renee Galloway, Aileen Tareg, Gabriela Paz-Bailey, Jorge L. Muñoz-Jordán, Tyler M. Sharp, Laura E. Adams, William A. Bower

**Affiliations:** ^1^Epidemic Intelligence Service, CDC; ^2^Division of High-Consequence Pathogens and Pathology, National Center for Emerging and Zoonotic Infectious Diseases, CDC; ^3^Yap State Department of Health Services, Federated States of Micronesia; ^4^Division of Vector-Borne Diseases, National Center for Emerging and Zoonotic Infectious Diseases, CDC.

On August 30, 2019, Yap State declared a public health crisis caused by concurrent outbreaks of dengue and leptospirosis, two clinically similar illnesses, resulting in 545 suspected dengue cases and 515 suspected leptospirosis cases during January–August 2019. Dengue virus type 3 (DENV-3) was identified by reverse transcription–polymerase chain reaction (RT-PCR) for 38 patients. Leptospirosis is endemic in Yap State (pop. approximately 11,500) with an anecdotal baseline of 0–2 cases/month.* Dengue is a potentially fatal mosquitoborne acute febrile viral illness (*1*). Leptospirosis is caused by infection with *Leptospira* bacteria and commonly acquired from contact with water or soil contaminated with infected animal urine (*2*). Approximately 90% of patients with leptospirosis experience self-limiting acute febrile illness, and 10% develop severe, potentially life-threatening illness (*3*). Early antibiotic treatment is generally associated with less severe and shorter illness (*4*). A team of outbreak investigators from Yap State and CDC identified suspected dengue and leptospirosis cases among patients with dengue-like illness (DLI),^†^ which is clinically compatible with both illnesses. The majority of patients with DLI were reported and tested as having suspected cases of both dengue and leptospirosis during the outbreak.

Among 515 patients with DLI tested for leptospirosis, 115 (22%) had a positive immunoglobulin M (IgM) leptospirosis rapid diagnostic test (LRDT) result.^§^ Because anti-*Leptospira* IgM antibodies can persist ≥12 months postinfection (*5*), the team performed testing to confirm the LRDT results and assess whether a leptospirosis outbreak occurred during the dengue outbreak.

During May–September 2019, the team collected paired acute-phase and convalescent-phase sera from patients^¶^ with DLI. Sera were tested for evidence of dengue by RT-PCR and enzyme-linked immunosorbent assay (ELISA)** at CDC and for leptospirosis by LRDT in Yap State and microscopic agglutination test (MAT), the serodiagnostic reference standard, at CDC. A laboratory-confirmed acute leptospirosis case was defined as a ≥fourfold increase in reciprocal MAT titers or any reciprocal MAT titer ≥800.^††^ The team calculated the LRDT’s performance characteristics and 95% confidence intervals (CIs).

Sera were tested from 103 patients, of whom 98 had paired sera. Forty-four patients (43%) tested positive for dengue by RT-PCR (40 patients) or ELISA (four). Five patients (5%) met the leptospirosis case definition; one patient seroconverted to a convalescent titer of 200, and four patients had titers ≥800 that did not change between acute and convalescent specimens, suggesting recent exposure (<6 months) but not acute infection. In addition, two of these four patients tested positive for DENV-3 by RT-PCR. An additional 11 patients (11%) had at least one titer ≥200 but <800 and lacked a ≥fourfold increase. Among 91 patients with LRDT and MAT results, 33 (36%) were LRDT-positive, including the five with confirmed cases (Table). The LRDT had a low positive predictive value of 15% (95% CI = 7%–31%) compared with MAT confirmation.

**TABLE:** Comparison of anti-*Leptospira* immunoglobulin M rapid diagnostic test (LRDT) results with leptospirosis microscopic agglutination test (MAT) results among patients with dengue-like illness* during a dengue outbreak — Yap State, Federated States of Micronesia, 2019

Result	MAT positive^†^	MAT negative^§^	Total
**LRDT positive**	5	28	33
**LRDT negative**	0	58	58
**Total**	**5**	**86**	**91**
**LRDT performance characteristic**		**%**	**(95% CI)**
Sensitivity		100	(57–100)
Specificity		67	(57–76)
Positive predictive value		15	(7–31)
Negative predictive value		100	(94–100)

**Abbreviation:** CI = confidence interval.

* Dengue-like illness was defined as fever with two or more of the following: nausea/vomiting, rash, aches and pains, any severe dengue warning sign (persistent vomiting, abdominal pain or tenderness, clinical fluid accumulation, mucosal bleeding, or lethargy/restlessness).

^†^ Criteria for a positive leptospirosis MAT result were a ≥4-fold increase in reciprocal MAT titers or any reciprocal MAT titer ≥800.

^§^ Results were considered MAT negative if there was a <4-fold increase in reciprocal MAT titers and all reciprocal titers were <800.

Testing performed at CDC confirmed the occurrence of a dengue outbreak in Yap State but did not support the occurrence of a leptospirosis outbreak because the number and frequency of confirmed cases were within the anecdotal baseline. The LRDT likely detected previous *Leptospira* exposures in most patients with positive results. During an outbreak of acute febrile illness in a leptospirosis-endemic area, health officials should consider the possibility of prolonged IgM detection when interpreting LRDTs. However, if leptospirosis is clinically suspected, antibiotic treatment should be initiated immediately. The recent experience in Yap State highlights the need for an antigen-based LRDT (i.e., alternative to antibody detection) and local capacity for PCR-based molecular diagnostics. Furthermore, the current case definition for laboratory-confirmed acute leptospirosis of any MAT titer ≥800 might not be appropriate for endemic areas because of the persistence of antibodies in the population.

## Footnotes

* The anecdotal baseline was based on hospital staff member recall (there is only one hospital in Yap) of the frequency of positive anti-*Leptospira* rapid diagnostic tests for patients with clinically compatible symptoms in the absence of a dengue outbreak.

† Dengue-like illness was defined as fever and two or more of the following: nausea/vomiting, rash, aches and pains, any severe dengue warning sign (persistent vomiting, abdominal pain or tenderness, clinical fluid accumulation, mucosal bleeding, or lethargy/restlessness).

§ SD Bioline *Leptospira* IgM, Abbott Laboratories. https://www.globalpointofcare.abbott/en/product-details/sd-bioline-leptospira.html.

¶ Patients were from Yap Memorial Hospital and four Wa’ab Community Health Center clinics in the Yap Main Islands.

** DENV Detect IgM Capture ELISA, InBios International. https://inbios.com/denv-detecttm-igm-capture-elisa-kit-intl/.

†† https://wwwn.cdc.gov/nndss/conditions/leptospirosis/case-definition/2013/.

## References

[1] World Health Organization. Dengue: guidelines for diagnosis, treatment, prevention and control. Geneva, Switzerland: World Health Organization; 2009. https://www.who.int/tdr/publications/documents/dengue-diagnosis.pdf 23762963

[2] CDC. Leptospirosis infection. Atlanta, GA: US Department of Health and Human Services, CDC; 2015. https://www.cdc.gov/leptospirosis/infection/index.html

[3] American Academy of Pediatrics. Leptospirosis. In: Kimberlin DW, Barady MT, Jackson MA, Long SS, eds. Red book: 2018 report of the Committee on Infectious Diseases. 31st ed. Itasca, IL: American Academy of Pediatrics; 2018:508–11.

[4] McClain JBL, Ballou WR, Harrison SM, Steinweg DL. Doxycycline therapy for leptospirosis. Ann Intern Med 1984;100:696–8. 10.7326/0003-4819-100-5-696 6712032

[5] Silva MV, Camargo ED, Batista L, Behaviour of specific IgM, IgG and IgA class antibodies in human leptospirosis during the acute phase of the disease and during convalescence. J Trop Med Hyg 1995;98:268–72. 7636924

